# Avoiding negativity bias: Towards a positive psychology of human–wildlife relationships

**DOI:** 10.1007/s13280-020-01394-w

**Published:** 2020-10-07

**Authors:** Arjen Buijs, Maarten Jacobs

**Affiliations:** 1 Forest and Nature Conservation Policy Group, Wageningen University, Droevendaalsesteeg 3, 6708 PG Wageningen, The Netherlands; 2 Cultural Geography Group, Wageningen University, Droevendaalsesteeg 3, 6708 PG Wageningen, The Netherlands

**Keywords:** Attitudes, Conservation, Human–wildlife interactions, Stewardship, Well-being, Wildlife

## Abstract

Recently, new approaches to wildlife management are being developed, such as coexistence management and convivial conservation. These approaches aim to shift management practices from mitigating human–wildlife conflicts towards cohabitation and explore mutual benefits. To align empirical research to these new approaches, we argue for the relevance of positive psychology theory to inspire and structure research into the benefits of human–wildlife interactions. Positive psychology suggests three pathways through which human–wildlife interactions may lead to happiness and well-being: pleasure, engagement, and meaning. Applying these pathways to human–wildlife research may (i) structure existing research into the benefits of human–wildlife interactions, (ii) disclose unidentified benefits of human–wildlife interactions, and (iii) unravel mechanisms which make experiencing and protecting wildlife worthwhile and rewarding. Also, we suggest a potential feedback loop between wildlife experiences, happiness and well-being, and pro-environmental behaviours. More in-depth research into these mechanisms may improve our understanding of attitudes towards conservation of wildlife and its habitat and may suggest strategies to strengthen stewardship actions and public support for conservation strategies. Together, these strands of research could initiate research into what could be called a “Positive Ecology”.

## Introduction

Human activities are the major driving force to influence wildlife populations through e.g. habitat fragmentation, hunting, poaching, or leisure practices and many other direct and indirect interactions (Woodroffe et al. 2005; Nyhus 2016). Over the years, strict conservation aims were formulated and humans and wildlife were separated in order to reduce negative effects of humans practices, including agriculture, leisure and hunting (Crespin and Simonetti 2019). Wildlife management practices have predominantly focused on habitat protection (Nepal and Weber 1995; Woodroffe et al. 2005; Redpath et al. 2015). Lethal control of wildlife posing a threat to human interests was often applied as management intervention, resulting in declining populations and even endangerment (Woodroffe et al. 2005).

Recently, new approaches to wildlife management are being developed, shifting from mitigating human–wildlife conflicts towards cohabitation of humans and wildlife (Woodroffe et al. 2005; Frank and Glikman 2019). Coexistence management, Convivial Conservation, and Nature-Based Thinking are proposed as new strategies to develop practices in which humans and wildlife inhabit the same landscape and can broadly satisfy their interests without posing severe mutual threats and competition for the same resources (Carter and Linnell 2016; Chapron and López-Bao 2016; Soulsbury and White 2016; Büscher and Fletcher 2019; Frank and Glikman 2019; Randrup et al. 2020). These approaches combine the normative ideal of cohabitation with a practical call to develop coexistence management approaches (Frank and Glikman 2019).

Despite an increased focus on coexistence management, empirical research on human–wildlife interactions still predominantly focuses on conflict situations and human tolerance of wildlife (e.g. Inskip et al. 2016, Soulsbury and White 2016, 2019). The extensive research focus on conflict and human tolerance in combination with limited and fragmentary research of the benefits is likely to over-emphasize the negative aspects of human–wildlife interactions (Soulsbury and White 2016). For example, in the USA, the concept of wildlife value orientations is widely applied to investigate social conflicts over wildlife in problem situations (Teel and Manfredo 2010). Psychological literature has a strong focus on tolerance and risk perceptions (Bruskotter and Wilson 2014) and research on emotions towards wildlife predominantly addresses fear towards large carnivores (Jacobs et al. 2014). These approaches have added to the understanding of human–wildlife interactions, yet they did not pay much attention to positive consequences of these interactions and are, thus, insufficiently equipped to underpin arguments for options and benefits of cohabitation of humans and wildlife.

A possible explanation for this focus on negative events and relationships is that a large portion of research has emerged from a field of study driven by management problems that require social science knowledge (Manfredo 2008; Bennett et al. 2017) and often funded by agencies experiencing those problems (Manfredo 2008). Subsequent negativity bias is a scientific problem if research suggests interactions with wildlife are more negative than they are. The same bias might hamper conservation success in wildlife management practices, due to blind spots for recognizing positive psychological opportunities, to be nurtured as sources for public support for wildlife conservation and coexistence management. The negativity bias translates into a knowledge need to recognize and comprehend potential positive interactions.

In this paper, we focus on the psychological benefits of human interactions with wildlife. Studies that empirically address positive effects of wildlife experiences do exist, focusing, for example, on the psychological benefits of experiences with wildlife (Curtin 2009; Soulsbury and White 2016), revenues via the recreation and tourism industry (Manfredo 2008), or contributions to conservation of wildlife and their habitats, for instance, through volunteering, donating, or membership (Treves and Martin 2011). Yet these studies are relatively few compared to research emphasizing problems. In addition, these studies are fragmented, with a strong focus on wildlife tourism and addressing a select few positive effects such as increased support for wildlife conservation (e.g. Curtin and Kragh 2014; McIntosh and Wright 2017; Bell et al. 2018). Taken together, research that focuses on positive psychological consequences of human–wildlife interactions does in our view not explore the full range of potential effects.

Human–wildlife interactions are not clearly defined in the literature, yet the concept is usually implicitly employed as an umbrella term covering any type of interaction (including seemingly one-directional interactions such as wildlife viewing). Attributes of interaction vary substantially in intensity, consequences, and frequency (Nyhus 2016; Soulsbury and White 2019). We define negative human–wildlife interactions here as the subset of interactions that cause problems of different types for either humans or wildlife or both (e.g. threats to safety, competing for resource). In general, negative interactions may include both human–wildlife conflicts such as threats to human health and safety (Manfredo 2008; Redpath et al. 2015) and human–human conflicts, such as controversy over wildlife management actions (Dickman 2010). We define positive human–wildlife interactions here as the subset of interactions that do not present substantial problems to either humans or wildlife and bring benefits to either humans or wildlife. Of course, whether an interaction is considered positive or negative partly depends on specific context and people’s interpretation of that context and is strongly related to economic and cultural backgrounds (Muhar et al. 2017). Especially between the Global South and the Global North, the context in which humans and wildlife interact differs. What works in the Global South may not work in the Global North and vice versa. In this paper, we focus on interactions among the Global North, where people are relatively wealthy and often do not experience severe conflicts with wildlife daily.

The aim of this perspective paper is to inspire a *positive psychology* of human–wildlife relationships and cohabitation. Empirical research within a framework of positive psychology can specify and substantiate benefits of coexistence approaches and provide and structure empirical evidence to the normative ideals of coexistence management. The next section introduces a theory from outside the environmental sciences that will be used to identify and structure known and potential positive effects and to suggest directions for future research: positive psychology theory.

## A positive psychology of human–wildlife relationships

Theories from positive psychology focus on understanding the factors that contribute to people’s well-being and happiness.^1^ In this paper, we define well-being as the subjectively experienced “capacity to be and do well in life, and achieve a state of health, happiness, or prosperity” (Russell et al. 2013, 474). Theory and research over the last 15 years has identified three pathways through which activities and experiences contribute to happiness and well-being: pleasure, engagement, and meaning (Table 1) (Seligman et al. 2004; Peterson et al. 2005; Seligman et al. 2005). *Pleasure* emerges through (i) experiencing positive emotions or (ii) avoiding negative emotions, including (iii) stress reduction. Psychological research has shown that although pleasure may contribute to happiness and well-being, this form of well-being is often short-lived and does not correlate to stable well-being as strongly as do engagement and meaning (Seligman et al. 2004). *Engagement* implies seeking gratification through (i) using and developing one’s virtues, personal growth and crafting one’s life, (ii) maintaining intimate relationships with family and friends, or (iii) experiencing flow by being fully immersed in activities during work or leisure (Ryff 1989; Seligman et al. 2006). The kind of activities, virtues, and intimate relationships depend on cultural and personal preferences and may range from being carried away by music to the daily care for family or animals. *Meaning* contributes to well-being through self-transcendence as the psychological result of i) belonging to something larger than oneself or ii) contributing to a valued purpose that is beyond one’s personal interests. Meaning places people’s life in a larger scheme and, thus, gives purpose to life (Ryff 1989; Howell et al. 2013).

**Table 1:** Pathways and mechanisms to well-being and happiness

	PATHWAYS		
	Pleasure	Engagement	Meaning
	Experiencing positive emotions	Flow experiences	Belonging
MECHANISMS	Avoiding negative emotions	Virtue development	Transcending personal interests
	Stress reduction	Intimate relationships	

In the next section, we will use the theoretical pathways and specific mechanisms to well-being represented in Table 1 for structuring and understanding the literature on benefits of experiencing wildlife or engaging in wildlife conservation projects as well as adjacent domains of research relevant to understanding human–wildlife interactions. For interpreting the literature, we make a distinction between (a) already existing evidence for mechanisms constituting positive human wildlife interactions, (b) evidence for mechanisms from adjacent research domains applicable, but yet untested to human–wildlife interactions, such as animal-assisted therapy (Kamioka et al. 2014), charity and volunteering behaviour (Jenkinson et al. 2013), or the benefits of nature experiences (e.g. Russell et al. 2013), and (c) identification and exploration of new mechanisms hitherto not or hardly examined.

## Benefits of human–wildlife relationships

We will now explore this existing evidence, suggestions from adjacent studies, and identification of yet unexplored mechanism, structured along the three pathways of human happiness: pleasure, engagement, and meaning.

### Pleasure

The majority of studies into benefits of human–wildlife interactions is related to the pleasure pathway. A positive psychology framework can theoretically structure exiting evidence on several well-established outcomes of human–wildlife interactions. One of the most established outcomes of previous research is the contribution of human–wildlife interactions on happiness through the positive feelings and emotions induced by wildlife encounters (Curtin 2009; Curtin and Kragh 2014; McIntosh and Wright 2017; Bell et al. 2018). Empirical evidence demonstrating that exposure to nature decreases stress (Hartig et al. 2014) can easily be translated to and applied in research into human–wildlife interactions. Applying well-established methods and theories from health studies will show whether similar impact of human–wildlife interactions on stress reduction exist, and whether these impact are similar or even bigger in magnitude than exposure to natural landscapes. In a similar vein, outcomes from pet research can be applied to study how being distracted by wildlife and enjoying its presence can eliminate a sense of isolation (Winefield et al. 2008). Furthermore, mechanisms suggested by positive psychology can inspire entirely new research questions, for example, through what mechanism decreasing negative feelings, such as stress and isolation, contributes to well-being, and how this operates in the context of human–wildlife interactions. In addition, how such mechanism may be used in a therapeutic context is an entirely new research question.

### Engagement

For the engagement pathway, positive psychology can also structure existing evidence, suggest how general psychological mechanisms can be explored in wildlife interactions, and also suggest new mechanisms to explore. For example, awe, fascination, and beauty have been identified as frequently occurring attributes of wildlife experiences (Curtin and Kragh 2014; Verma et al. 2015). Positive psychology theory posits that these experiential attributes can constitute experiences of flow. This inspires new research questions on the relationship between experiencing wildlife and flow. Such research would examine how human–wildlife interactions can contribute to long-term well-being through frequently experiencing flow through being fully immersed in the search for wildlife (e.g. birdwatchers, wildlife photographers) (Kruger et al. 2015). For the engagement pathway, positive psychology suggests that intimate relationships between individuals and human communities contribute to well-being. Pet research suggests that relationships with animals can have similar effects (Winefield et al. 2008). It would be worth investigating how prolonged contact with, interest in or care for wild animals, specific species and their habitats relationships contribute to happiness through the engagement pathway. Interesting examples include bird feeding in people’s gardens, species monitoring, or birding activities (Horn and Johansen 2013; Raymond et al. 2018; Ganzevoort and Van Den Born 2019). Human–wildlife relationships also foster engagement indirectly as the presence of specific types of wildlife in familiar places —leisure, residential, or work-related places— contribute to sense of place, including feelings of identity (e.g. wildlife as totem denoting communities) (Davenport and Anderson 2005; Jacobs and Buijs 2011; Folmer et al. 2016). Finally, positive psychology theory suggests a new strand of research, to examine if developing personal strengths, such as acquiring knowledge about wildlife or increasing skills on wildlife tracking, would be emotionally rewarding for an individual.

### Meaning

The happiness mechanism via meaning —the third pathway in positive psychology— is probably the one that is least explored in research into human–wildlife relationships. Spiritual meanings assigned to nature in general and to wildlife specifically are frequently investigated, as well as ego-transcendence resulting from wildlife encounters (Vidon et al. 2018; Verschuuren and Brown 2019). However, the benefits people derive from these meanings through (re)connecting to something larger than oneself are not yet empirically addressed in human–wildlife research. Next to being part of something larger than oneself, also the idea of “doing good”, that is, actions based on altruism, make people feel happy (Bechtel and Corral Verdugo 2010; Van den Born et al. 2018). Indeed, volunteering for wildlife or habitat conservation has been shown to contribute to the meaning pathway towards well-being (Corral Verdugo 2012). This may also stem from the satisfaction of leaving a legacy to wildlife and/or future generations (Raymond et al. 2018). Developing this rather unexplored mechanism to happiness may suggest additional pathways through which human–wildlife relationships contribute to people’s well-being.

Table 2 illustrates some of the examples of how a positive psychology framework may structure existing research and inspire new research through translating proven psychological mechanisms or suggesting new mechanisms to investigate in benefits of human–wildlife interactions.

**Table 2:** Framework for investigating HWI through a positive ps framework: To structure, apply, and explore evidence for contributions of HWI to well-being and happiness for each pathway of positive psychology, with examples

	PATHWAYS		
	Pleasure	Engagement	Meaning
STRUCTURE EXISTING EVIDENCE	Experiencing wildlife evokes positive feelings	Experiencing awe, fascination, and beauty Experiencing relationships with wildlife	Spiritual meanings
APPLY PROVEN MECHANISMS FROM OTHER FIELDS	Impact of HWI on stress reduction	Wildlife contributing to sense of place	Connecting to something larger Doing good
EXPLORE NEW MECHANISMS	Decrease of negative feelings through HWI Therapeutic practices	Flow of being fully immersed in search for wildlife	–

## Consequences for conservation practices

We have focused thus far on the contribution of human–wildlife relationships to human well-being and happiness. However, several studies suggest an additional causal relationship: happiness may stimulate pro-conservation attitudes and positive conservation behaviours (Ballantyne et al. 2011; Corral Verdugo 2012). Happier people more often engage in environmental stewardship practices and emotional affinity with nature is an important motivator to protect nature (Kals et al. 1999). This link between contributing to the protection of wildlife and living a meaningful life suggests that especially the meaning pathway to happiness contributes to positive conservation attitudes and active stewardship (Raymond and Raymond 2019).

**Fig. 1:**
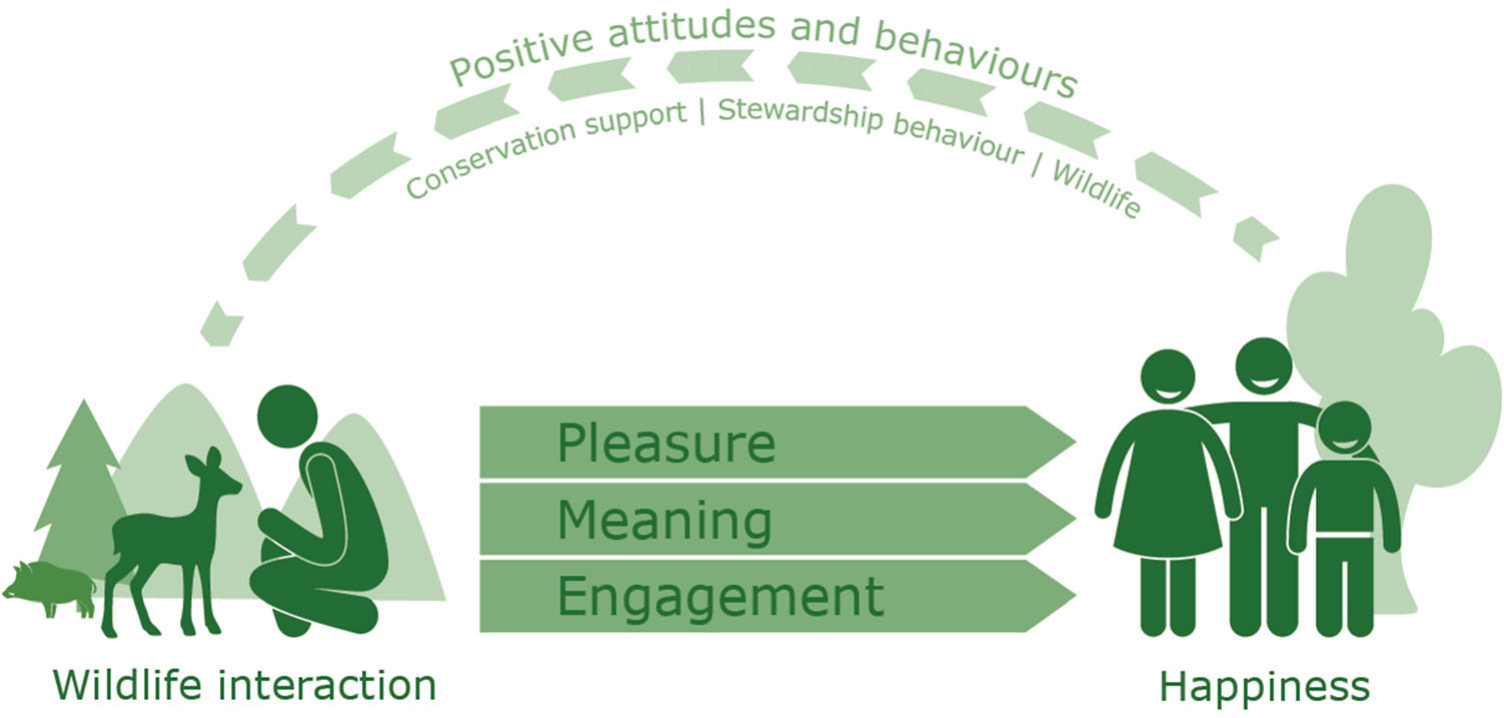
A positive psychology view on human–wildlife interactions

Based on this still rather limited research, we stipulate that a positive feedback loop may exist between happiness and positive attitudes and behaviours, including wildlife-focused stewardship practices: positive human–wildlife interactions and acting upon it through stewardship practices may lead to increased pleasure, meaning, and engagements in the context of wildlife. Subsequent increases in happiness and well-being may lead to increased positive wildlife conservation attitudes and behaviour, which may contribute to protecting and increasing wildlife populations and, thus, allow for more positive experiences (see Fig. 1). Empirical research is needed to provide evidence for this suggested positive feedback loop. Studies in European landscapes inhabited by brown bears and humans suggest that positive experiences foster coexistence (Majić et al. 2011; Dorresteijn et al. 2016).

## Discussion and conclusions

In this perspective paper, we have argued that a positive psychology framework fosters systematic identification, understanding, and future examination of human–wildlife interactions that contribute to human happiness. Broader and more comprehensive research on positive human–wildlife interactions better aligns with contemporary wildlife management practices focusing on coexistence rather than on conflict exclusively.

Coexistence with wildlife is more than living together in the same landscape through avoiding conflict. Nurturing positive human–wildlife interactions is at least equally important. Balanced understanding of human–nature relationships and more systematic understanding of positive consequences of human–wildlife interactions would complement the current knowledge base focusing on negative consequences. A more balanced knowledge base can be useful for conservation practices through understanding pathways to more positive conservation attitudes and behaviours of the general public as well as specific target groups (Raymond and Raymond 2019). Eventually, this could result in a focus on “Positive Ecology” research (Cf. Schmidt 2018), focusing on pleasure, engagement, and meaning.

To increase positive attitudes toward nature, wildlife, and pro-conservation behaviour, awareness of negativity bias by policy makers and managers is conducive. As an example, the Dutch NGO Living with Wolves (*Leven met Wolven*) aims to foster coexistence between wolves and humans in The Netherlands (Wolven-in-Nederland 2020). Communication with the Dutch population is an important strategy. On the page devoted to humans and wolves, human fear for wolves is extensively mentioned and explanations tell the visitor that fear is not needed. Yet, by focusing on negative emotions, the potential of nurturing positive emotions to foster coexistence remains locked. This is the more problematic as research suggests that fear for wolves is hardly associated with support for lethal control, while positive emotions such as joy and interest are associated with less support for lethal control (Jacobs et al. 2014). Using the positive psychology framework would prevent this blind spot by anticipating the potential of positive experiences.

Deliberately or unreflectively, conservation practices already partially focus on activities that contribute to happiness, meaning, or engagements. Providing onsite-opportunities for wildlife experience is an example of contributing to happiness through human–wildlife encounters. However, through a more explicit focus on the less well-known pathways of meaning and engagement, conservationists may make further contributions to positive experiences and happiness. In addition, onsite engagement in conservation practices could expand the focus from knowledge transfer to engagement and meanings to stimulate pro-conservation behaviours. Human behaviour is inherently complex and consequently drivers and motivations to support conservation policies and to actively engage in conservation and stewardship practices are many (Buijs et al. 2018, 2019; Pagès et al. 2018; Van den Born et al. 2018). Positive experiences are one of the possible drivers (Hartig et al. 2001). The link with positive experiences proposed in our framework may inspire the search for additional motivating factors.

Positive psychology provides a theoretical basis for this endeavour. As this paper illustrated, application of the mechanisms to well-being in the specific context of human–wildlife interactions is useful to interpret existing research and identify positive consequences not yet taken into account. The resulting positive psychology of human–wildlife interactions could also be extended to other types of positive human–nature interactions, such as health benefits of nature, natural landscape experiences, or biodiversity exposure. Together, these strands of research could initiate what could be called a “Positive Ecology”.

## Footnotes

1 However, definitions and conceptualisations of subjective well-being (as well as happiness) are highly debated in literature (e.g. Ryff 1989; Seligman and Csikszentmihalyi 2000; Peterson et al. 2005; Corral Verdugo 2012; Howell et al. 2013). In this paper we will use the term happiness and (subjective) well-being interchangeably.
